# Low Adiponectin Levels Are an Independent Predictor of Mixed and Non-Calcified Coronary Atherosclerotic Plaques

**DOI:** 10.1371/journal.pone.0004733

**Published:** 2009-03-06

**Authors:** Uli C. Broedl, Corinna Lebherz, Michael Lehrke, Renee Stark, Martin Greif, Alexander Becker, Franz von Ziegler, Janine Tittus, Maximilian Reiser, Christoph Becker, Burkhard Göke, Klaus G. Parhofer, Alexander W. Leber

**Affiliations:** 1 Department of Internal Medicine II, University of Munich, Munich, Germany; 2 Department of Internal Medicine I, University of Munich, Munich, Germany; 3 Helmholtz-Zentrum, Munich, Germany; 4 Department of Radiology, University of Munich, Munich, Germany; University of Pennsylvania, United States of America

## Abstract

**Background:**

Atherosclerosis is the primary cause of coronary artery disease (CAD). There is increasing recognition that lesion composition rather than size determines the acute complications of atherosclerotic disease. Low serum adiponectin levels were reported to be associated with coronary artery disease and future incidence of acute coronary syndrome (ACS). The impact of adiponectin on lesion composition still remains to be determined.

**Methodology/Principal Findings:**

We measured serum adiponectin levels in 303 patients with stable typical or atypical chest pain, who underwent dual-source multi-slice CT-angiography to exclude coronary artery stenosis. Atherosclerotic plaques were classified as calcified, mixed or non-calcified. In bivariate analysis adiponectin levels were inversely correlated with total coronary plaque burden (r = −0.21, p = 0.0004), mixed (r = −0.20, p = 0.0007) and non-calcified plaques (r = −0.18, p = 0.003). No correlation was seen with calcified plaques (r = −0.05, p = 0.39). In a fully adjusted multivariate model adiponectin levels remained predictive of total plaque burden (estimate: −0.036, 95%CI: −0.052 to −0.020, p<0.0001), mixed (estimate: −0.087, 95%CI: −0.132 to −0.042, p = 0.0001) and non-calcified plaques (estimate: −0.076, 95%CI: −0.115 to −0.038, p = 0.0001). Adiponectin levels were not associated with calcified plaques (estimate: −0.021, 95% CI: −0.043 to −0.001, p = 0.06). Since the majority of coronary plaques was calcified, adiponectin levels account for only 3% of the variability in total plaque number. In contrast, adiponectin accounts for approximately 20% of the variability in mixed and non-calcified plaque burden.

**Conclusions/Significance:**

Adiponectin levels predict mixed and non-calcified coronary atherosclerotic plaque burden. Low adiponectin levels may contribute to coronary plaque vulnerability and may thus play a role in the pathophysiology of ACS.

## Introduction

Atherosclerosis is the primary cause of coronary artery disease (CAD), one of the most common causes of illness and death worldwide. There is increasing recognition that lesion composition rather than size determines the acute complications of atherosclerotic disease in humans. Several studies suggested that thin-cap fibroatheroma (non-obstructive plaques) are prone to rupture and result in acute coronary artery occlusions [1]–[3], whereas obstructive, calcified plaques result in clinically stable angina pectoris.

Initiation and progression of the atherosclerotic lesion are highly complex processes, and many aspects of atherogenesis remain incompletely understood. Ectopic visceral adipose tissue was linked to the pathogenesis of atherosclerosis due to secretion of a multitude of pro- and anti-atherogenic cytokines and adipokines [4]. More recently, pericardial adipose tissue (PAT) was also reported to play an important role in coronary atherosclerosis [5], presumably through paracrine and vasocrine signaling of adipokines [6].

Adiponectin is the most abundant adipokine produced by adipose tissue. Serum levels of adiponectin are markedly decreased in patients with visceral obesity and states of insulin resistance such as non-alcoholic fatty liver disease and type 2 diabetes [7], [8]. There is an ongoing debate regarding adiponectin's significance for CAD. Although experimental data do suggest an atheroprotective effect [9], existing epidemiological data connecting adiponectin and cardiovascular disease are controversial. Low adiponectin levels have been linked to the presence of CAD [10] and were shown to be a risk factor for CAD [11] and cardiovascular events [12]. Low adiponectin levels were reported to be associated with a higher risk of acute coronary syndrome, independent of other traditional metabolic and cardiovascular risk factors [13]. Low adiponectin levels were also reported to be associated with the progression of coronary artery calcification as determined by electron-beam CT [14]. Furthermore, low serum adiponectin levels were shown to be an independent predictor of the extent of CAD and coronary lesion complexity as determined by coronary angiography [15]–[17]. In contrast, other studies including a recent meta-analysis of 7 prospective reports on adiponectin and coronary heart disease in Western populations failed to show an association between adiponectin and incident coronary heart disease as well as secondary cardiovascular events in patients with known CAD [18], [19].

The angiographic assessment of coronary luminal stenosis has been considered a surrogate marker of the severity of atherosclerosis. However, coronary angiography has low predictive value to assess atherosclerotic plaque burden or to predict acute coronary syndrome events [20]–[22]. It does not allow to identify non-obstructive coronary plaques or to determine the composition of atherosclerotic plaques. Therefore, in the present study, we used dual-source multi-slice CT (DSCT)-angiography to quantitatively and qualitatively assess coronary artery plaques to test whether 1) adiponectin serum levels are associated with coronary atherosclerotic plaque burden and 2) adiponectin levels are associated with coronary atherosclerotic plaque morphology.

## Results

Baseline characteristics of the study population are shown in Table 1. 303 consecutive patients with stable typical or atypical chest pain underwent DSCT-coronary angiography to exclude coronary artery stenosis.

**Table 1:** Baseline characteristics and CT-angiographic findings of the study population.

Characteristics		n = 303
Age (yrs)		63 (55–70)
Sex	male	202
	female	101
Body mass index (kg/m^2^)		26.2 (24.1–29.0)
Hypertension*	yes	147
	no	126
Diabetes mellitus*	yes	20
	no	253
Smoker†	yes	42
	no	230
Family history of CAD†	yes	77
	no	195
Laboratory profile	LDL-cholesterol (mg/dl)	122 (95–149)
	HDL-cholesterol (mg/dl)	52 (44–59)
	Triglycerides (mg/dl)	143 (106–207)
	hsCRP (mg/dl)	0.23 (0.05–0.54)
	Adiponectin (µg/ml)	5.1 (3.3–7.8) (range 0.2–23.4)
Medical treatment‡	Statin	120
	Asa, Plavix or Marcumar	170
	Betablocker	173
	ACE-I or ARB	138
	Diuretics	87
	Insulin or OAD	16
Pericardial adipose tissue volume (ml)§		190 (132–259)
Number of coronary artery plaques (total)∥		3 (1–6) (range 0–26)
Number of calcified plaques		1 (0–3) (range 0–22)
Number of mixed plaques		0 (0–1) (range 0–10)
Number of non-calcified plaques		0 (0–2) (range 0–9)

Values are presented as n or median (interquartile range).

* History of diabetes and hypertension is known in 273 patients.

† History of smoking and family history of CAD is known in 272 patients.

‡ Medication is known in 258 patients.

§ Adequate image quality for evaluation of PAT volume was obtained in 287 patients.

∥ Adequate image quality for evaluation of coronary plaques was obtained in 281 patients.

In 60 patients no coronary plaques could be detected. The median number of coronary plaques was 3 (IQR: 1–6; range: 0–26). The median numbers of calcified, mixed and non-calcified plaques were 1 (IQR: 0–3; range: 0–22, number of patients with calcified plaques: 154), 0 (IQR: 0–1; range: 0–10, number of patients with mixed plaques: 102) and 0 (IQR: 0–2; range: 0–9, number of patients with non-calcified plaques: 126), respectively.

### Correlations and associations between adiponectin and cardiovascular risk factors

Serum adiponectin levels were positively correlated with HDL-cholesterol (HDL-C) (r = 0.32, p<0.0001) and age (r = 0.16, p = 0.005), yet negatively correlated with body mass index (BMI) (r = −0.26, p<0.0001) and triglycerides (r = −0.32, p<0.0001) (Table 2). Interestingly, adiponectin was negatively correlated with PAT volume (r = −0.24, p<0.0001). No correlation was seen with LDL-cholesterol (LDL-C) (r = 0.09, p = 0.11) and high sensitivity C-reactive protein (hsCRP) levels (r = −0.04, p = 0.49).

**Table 2:** Correlations and associations between adiponectin and cardiovascular risk factors.

Characteristics			p
Age		0.16	0.005
Sex	Male	4.7 (2.9–6.6)	<0.0001
	Female	6.9 (4.1–10.1)	
Body mass index (kg/m^2^)		−0.26	<0.0001
Actual hypertension	Yes	5.0 (2.9–7.5)	0.13
	No	5.1 (3.4–8.1)	
History of diabetes mellitus	Yes	4.2 (2.2–8.3)	0.37
	No	5.0 (3.1–7.7)	
Actual smoker	Yes	3.9 (2.4–5.7)	0.02
	No	5.3 (3.2–7.9)	
Family history of CAD	Yes	4.3 (2.5–7.5)	0.05
	No	5.3 (3.4–7.9)	
LDL-cholesterol (mg/dl)		0.09	0.11
HDL-cholesterol (mg/dl)		0.32	<0.0001
Triglycerides (mg/dl)		−0.32	<0.0001
High-sensitivity CRP (mg/dl)		−0.04	0.49
Pericardial adipose tissue (ml)		−0.24	<0.0001
Total number of coronary artery plaques		−0.21	0.0004
Number of calcified plaques		−0.05	0.39
Number of mixed plaques		−0.20	0.0007
Number of non-calcified plaques		−0.18	0.003

Values are presented as correlation coefficient or median (interquartile range).

Age (r = 0.38, p<0.0001) and HDL-cholesterol (r = −0.21, p = 0.0003) as internal controls of our data were significantly correlated with total plaque burden in bivariate analysis.

Among demographic characteristics, smoking (p = 0.02) was associated with lower adiponectin levels. No or borderline association was seen with diabetes mellitus (p = 0.37), hypertension (p = 0.13), and family history of CAD (p = 0.05).

### Adiponectin is an independent negative predictor of coronary atherosclerotic burden

In bivariate analysis, adiponectin levels were negatively correlated with total number of coronary artery plaques (r = −0.21, p = 0.0004) (Table 2). In a fully adjusted multivariate model containing age, sex, BMI, hypertension, diabetes mellitus, smoking, family history of CAD, LDL-C, HDL-C, triglycerides, hsCRP, medication and PAT volume, adiponectin levels were predictive of coronary atherosclerotic plaque burden, i.e. an increase in adiponectin levels by 27.8 µg/ml is associated with the reduction of one atherosclerotic plaque (estimate: −0.036, 95%CI: −0.052 to −0.020, p<0.0001) (Table 3). Thus, based on the range of adiponectin and the range of total plaque number, adiponectin levels account for approximately 3% of the variability in total plaque burden in our study population.

**Table 3:** Multivariate association of adiponectin levels with coronary atherosclerotic plaque burden.

	Coronary atherosclerotic plaque burden		
	estimate	95% CI	p
Model 1*	−0.057	−0.072 to −0.043	<0.0001
Model 2†	−0.048	−0.063 to −0.033	<0.0001
Model 3‡	−0.041	−0.056 to −0.025	<0.0001
Model 4§	−0.037	−0.053 to −0.021	<0.0001
Model 5∥	−0.036	−0.052 to −0.020	<0.0001

Estimate and 95% CI for decrease in the number of total coronary atherosclerotic plaques for an 1 µg/ml increase in serum adiponectin levels. Full model 5 (including all parameters) is shown in Table S1 of the supplement.

* adjusted for age, sex and BMI.

† adjusted for age, sex, BMI, hypertension, diabetes mellitus, smoking and family history of CAD.

‡ adjusted for age, sex, BMI, hypertension, diabetes mellitus, smoking, family history of CAD, LDL-C, HDL-C, triglycerides and hsCRP levels.

§ adjusted for age, sex, BMI, hypertension, diabetes mellitus, smoking, family history of CAD, LDL-C, HDL-C, triglycerides, hsCRP levels and medication.

∥ adjusted for age, sex, BMI, hypertension, diabetes mellitus, smoking, family history of CAD, LDL-C, HDL-C, triglycerides, hsCRP levels, medication and PAT volume.

### Adiponectin is inversely associated with mixed and non-calcified coronary artery plaques

Adiponectin was negatively correlated with mixed (r = −0.20, p = 0.0007) and non-calcified plaques (r = −0.18, p = 0.003) (Table 2). No correlation was seen with calcified plaques (r = −0.05, p = 0.39). In fully adjusted multivariate models, adiponectin levels remained a negative predictor of mixed and non-calcified plaque burden. Thus, the number of mixed plaques decrease by 1 for an 11.5 µg/ml increase in serum adiponectin levels (estimate: −0.087, 95%CI: −0.132 to −0.042, p = 0.0001) (Table 4). Furthermore, an increase in adiponectin levels by 13.2 µg/ml is associated with the reduction of one non-calcified plaque (estimate: −0.076, 95%CI: −0.115 to −0.038, p = 0.0001) (Table 5). Based on the range of adiponectin and the range of mixed and non-calcified plaque numbers, adiponectin levels account for approximately 20% of the variability in mixed and non-calcified plaque burden.

**Table 4:** Multivariate association of adiponectin levels with mixed plaques.

	Mixed plaques		
	estimate	95% CI	p
Model 1*	−0.090	−0.127 to −0.053	<0.0001
Model 2†	−0.078	−0.115 to −0.040	<0.0001
Model 3‡	−0.080	−0.120 to −0.041	<0.0001
Model 4§	−0.083	−0.126 to −0.039	0.0002
Model 5∥	−0.087	−0.132 to −0.042	0.0001

Estimate and 95% CI for decrease in the number of mixed plaques for an 1 µg/ml increase in serum adiponectin levels. Full model 5 (including all parameters) is shown in Table S2 of the supplement.

* adjusted for age, sex and BMI.

† adjusted for age, sex, BMI, hypertension, diabetes mellitus, smoking and family history of CAD.

‡ adjusted for age, sex, BMI, hypertension, diabetes mellitus, smoking, family history of CAD, LDL-C, HDL-C, triglycerides and hsCRP levels.

§ adjusted for age, sex, BMI, hypertension, diabetes mellitus, smoking, family history of CAD, LDL-C, HDL-C, triglycerides, hsCRP levels and medication.

∥ adjusted for age, sex, BMI, hypertension, diabetes mellitus, smoking, family history of CAD, LDL-C, HDL-C, triglycerides, hsCRP levels, medication and PAT volume.

**Table 5:** Multivariate association of adiponectin levels with non-calcified plaques.

	Non-calcified plaques		
	estimate	95% CI	p
Model 1*	−0.091	−0.126 to −0.057	<0.0001
Model 2†	−0.082	−0.117 to −0.048	<0.0001
Model 3‡	−0.082	−0.120 to −0.044	<0.0001
Model 4§	−0.074	−0.112 to −0.036	0.0001
Model 5∥	−0.076	−0.115 to −0.038	0.0001

Estimate and 95% CI for decrease in the number of non-calcified plaques for an 1 µg/ml increase in serum adiponectin levels. Full model 5 (including all parameters) is shown in Table S3 of the supplement.

* adjusted for age, sex and BMI.

† adjusted for age, sex, BMI, hypertension, diabetes mellitus, smoking and family history of CAD.

‡ adjusted for age, sex, BMI, hypertension, diabetes mellitus, smoking, family history of CAD, LDL-C, HDL-C, triglycerides and hsCRP levels.

§ adjusted for age, sex, BMI, hypertension, diabetes mellitus, smoking, family history of CAD, LDL-C, HDL-C, triglycerides, hsCRP levels and medication.

∥ adjusted for age, sex, BMI, hypertension, diabetes mellitus, smoking, family history of CAD, LDL-C, HDL-C, triglycerides, hsCRP levels, medication and PAT volume.

After adjusting for traditional cardiovascular risk factors adiponectin turned out to be significantly associated with calcified plaques (Table 6). In a fully adjusted model, however, this association was lost again (estimate: −0.021, 95%CI: −0.043 to 0.001, p = 0.06).

**Table 6:** Multivariate association of adiponectin levels with calcified plaques.

	Calcified plaques		
	estimate	95% CI	p
Model 1*	−0.044	−0.064 to −0.024	<0.0001
Model 2†	−0.039	−0.060 to −0.019	0.0001
Model 3‡	−0.026	−0.047 to −0.005	0.01
Model 4§	−0.022	−0.044 to −0.000	0.05
Model 5∥	−0.021	−0.043 to 0.001	0.06

Estimate and 95% CI for decrease in the number of calcified plaques for an 1 µg/ml increase in serum adiponectin levels. Full model 5 (including all parameters) is shown in Table S4 of the supplement.

* adjusted for age, sex and BMI.

† adjusted for age, sex, BMI, hypertension, diabetes mellitus, smoking and family history of CAD.

‡ adjusted for age, sex, BMI, hypertension, diabetes mellitus, smoking, family history of CAD, LDL-C, HDL-C, triglycerides and hsCRP levels.

§ adjusted for age, sex, BMI, hypertension, diabetes mellitus, smoking, family history of CAD, LDL-C, HDL-C, triglycerides, hsCRP levels and medication.

∥ adjusted for age, sex, BMI, hypertension, diabetes mellitus, smoking, family history of CAD, LDL-C, HDL-C, triglycerides, hsCRP levels, medication and PAT volume.

## Discussion

Our study was designed to examine the relationship of adiponectin with total coronary atherosclerotic plaque burden and atherosclerotic plaque morphology in humans. We demonstrate that 1) adiponectin levels are predictive of total coronary plaque burden, and 2) adiponectin levels are inversely correlated with the number of mixed and non-calcified plaques. No significant correlation was seen with calcified plaques. Our data suggest that the number of mixed and non-calcified plaques decrease by 1 for each 11.5 µg/ml and 13.2 µg/ml increase in serum adiponectin levels, respectively.

Pundziute *et al.* [23] reported that the number of coronary segments with mixed plaques as determined by MSCT was an independent predictor of acute cardiac events including cardiac death, nonfatal myocardial infarction and unstable angina requiring hospitalization. Hoffmann *et al.* [24] demonstrated that in contrast to calcified plaques, non-calcified plaques as determined by MSCT were consistently present in culprit lesions of patients with acute coronary syndrome. We have recently shown that rapid progression of angiographic stenosis severity occurred most frequently in coronary segments with non-calcified or predominantly non-calcified plaques as determined by MSCT [25]. Wolk *et al.* [13] reported that higher plasma adiponectin levels were associated with a lower risk of acute coronary syndrome, suggesting that the pathophysiological role of adiponectin may be related to the stability of atherosclerotic plaque rather than atherosclerotic burden. Our data indicate that adiponectin may reduce the number of non-calcified and mixed plaques without affecting stable calcified plaques. Consistent with these observations, Marso *et al.* [26] showed in a very recent study that low adiponectin levels were associated with lipid-rich yet not calcified coronary plaques in non-diabetic patients in bivariate analysis as determined by IVUS. Furthermore, Otake *et al.* [27] demonstrated that the necrotic core component ratio of culprit lesions as determined by IVUS in patients with acute coronary syndrome was negatively correlated with adiponectin levels in bivariate analysis. Therefore, the finding of an independent association between adiponectin and non-calcified and mixed coronary plaques may have important therapeutic and preventive implications for decreasing the risk of acute coronary events.

Although the exact mechanisms of the negative association between adiponectin and mixed and non-calcified plaques remain to be determined, several possible explanations should be considered. In addition to its beneficial effects on insulin sensitivity and lipid metabolism [28], adiponectin exerts its vasculoprotective effects through its direct actions on endothelial cells, monocytes, macrophages and other inflammatory cells, platelets and smooth muscle cells, thus modulating initiation and progression of atherosclerosis. Adiponectin augments endothelial NO production [29], [30], inhibits ox-LDL–induced endothelial ROS generation [31], suppresses the expression of endothelial adhesion molecules [32]–[34], attenuates leucocyte-endothelium interactions [35] and protects endothelial cells from apoptosis [36]. Furthermore, adiponectin inhibits macrophage activation [37], [38] and foam cell formation [39], promotes the clearance of early apoptotic cells by macrophages [40], inhibits smooth muscle cell proliferation [32], [41] and antagonizes the stimulatory effect of TNF-α on vascular smooth muscle cell calcification [42].

The present study has several strengths and limitations. Our data support the concept that adiponectin is an important marker in the pathogenesis of atherosclerosis since adiponectin remains significantly associated with plaque morphology in a fully adjusted multivariate model containing age, sex, BMI, hypertension, diabetes mellitus, smoking, family history of CAD, LDL-C, HDL-C, triglycerides, hsCRP, medication and PAT volume. However, since this was an association study, our study does not establish a causal relationship between adiponectin and coronary plaque morphology. Furthermore, the clinical implications of our results still need to be determined.

There are conflicting epidemiological data regarding the role of adiponectin in atherosclerosis with some studies showing strong inverse associations between adiponectin levels and CAD [10]–[17] and others failing to detect any association [18], [19]. Differences in study design, population characteristics and statistical adjustments may in part explain the apparent discrepancies. In addition, different methods of CAD assessment (CT-angiography or IVUS versus conventional angiography or CT-based assessment of coronary artery calcification) may yield distinct results. Adiponectin may modulate early stages of atherogenesis thus mainly affecting the development of non-calcified or mixed plaques.

In the present study adiponectin is not correlated with calcified plaques, the majority of coronary plaques in our study cohort. Therefore, although statistically highly significant, adiponectin levels are associated with a very modest decrease in total coronary plaque burden accounting for only 3% of the variability in total number of coronary plaques. In contrast, adiponectin accounts for approximately 20% of the variability in mixed and non-calcified plaque burden. Determination of the bioactive high molecular weight form of adiponectin may have possibly resulted in an even stronger association of adiponectin with plaque burden and morphology. Overall, our data emphasize the importance of additional risk factors in the pathogenesis of atherosclerosis.

Storage conditions of blood samples (e.g. storage time and temperature) were suggested to alter adiponectin levels [43], [44]. However, comparing adiponectin concentrations in serum aliquots stored for 21 and 33 months at −70°C, adiponectin levels were not significantly different (p = 0.45) suggesting that storage conditions did not impact on our results due to adequate specimen stability.

Finally, our study results may only be applicable to non-diabetic patients. Diabetics were clearly underrepresented in the present study (7% of patients). The small number of diabetic patients may also explain the lack of association between adiponectin levels and diabetes.

In summary, we demonstrate that adiponectin is inversely associated with coronary plaque burden and mixed and non-calcified plaques, suggesting an important role of adiponectin in the pathogenesis of atherosclerosis and possibly in the pathophysiology of acute coronary syndrome.

## Methods

### Ascertainment of Subjects

303 consecutive patients who underwent DSCT-coronary angiography for exclusion of coronary artery stenosis due to stable typical or atypical chest pain, were recruited during 20 consecutive months from March 2006 to October 2007. After providing informed written consent, study subjects were asked to complete a brief questionnaire and have blood drawn. The study protocol was approved by the Ethics Committee of the Ludwig-Maximilians-University Munich, Germany.

### Dual-source multi-slice CT-coronary angiography

CT-coronary angiography was performed using a Siemens Definition scanner (Siemens Medical Solutions, Forchheim, Germany) that uses two X-ray sources for image generation. Tube voltage for CT-angiography was 120 kV for both tubes in patients with a body weight >80 kg and 100 kV for those with a weight <80 kg. Current was 560 mA with modulation, and full current between 30–50% to 80% of the cardiac cycle. Gantry rotation time was 0.33 s, and pitch 0.2–0.44 adapted to the HR. Per rotation 64 slices were generated with a collimation of 0.6 mm, leading to an isotropic voxel resolution of approximately 0.6 mm edge length and 0.2 mm^3^ volume. Before the scan, nitroglycerine was administered sublingually. A bodyweight-adapted volume of contrast agent (1.25 cm^3^/kg bodyweight, Ultravist 370, Schering, Berlin, Germany) was injected continuously at a calculated rate to achieve constant injection during 20 s. The scan was started with a delay of 5 s after the density in the aortic root exceeded a density value of 100 HU (bolus tracking). A saline flush (100 cm^3^ at 5 cm^3^/s) was applied to maintain a compact bolus. Axial images were reconstructed with 0.75 mm slice thickness and 0.5 mm increment using a medium sharp convolution kernel (B26f) and retrospective ECG gating. The reconstructions were performed in 10% steps over the entire R-R cycle using a single-segment algorithm that utilizes a quarter segment of projection data from both detectors. In atrial fibrillation, data were reconstructed in 50 ms steps.

### Dual-source CT image analysis

#### Coronary analysis

In the first step, all reconstructed data sets were evaluated at different ECG-phases for diagnostic image quality and the optimal data set was then chosen for analysis. The DSCT datasets were evaluated by two independent investigators blinded to serum adiponectin levels using a dedicated cardiac workstation (Siemens, Leonardo Circulation).

Atherosclerotic plaques were classified as calcified, mixed or non-calcified as described previously [45]. Calcified plaques were defined as lesions with a HU value above 130. Non-calcified plaques were defined as structures clearly assignable to the vessel wall (in at least two views) with densities less than the lumen contrast. Plaques with <50% calcified plaque area were classified as mixed. The coronary tree was segmented according to the suggestions of the AHA into a 15 segment model. Each segment was further divided into a proximal and a distal segment. Each segment was then classified as containing either calcified, non-calcified, mixed or no plaque. Based on the number of diseased segments a plaque score was calculated. Adequate image quality for evaluation of coronary plaques was obtained in 281 of 303 patients.

The interobserver agreement of the 2 investigators was 95% for calcified plaques, 94% for mixed plaques, and 93% for non-calcified plaques.

#### Pericardial fat assessment

The same images as for the analysis of atherosclerotic plaques were used to determine PAT volume. PAT volume was measured in ml using the volume analysis software tool of the Siemens Leonardo Circulation workstation. PAT volume was determined similar to the method described by Gorter et al. [46]. We defined PAT as the adipose tissue surrounding the myocardium. The upper cut off point in the axial slices was the bifurcation of the pulmonary artery. Adequate image quality for evaluation of PAT volume was obtained in 287 of 303 patients. The interobserver agreement for PAT volume was 95%.

### Laboratory procedures

Blood samples were stored in aliquots at −70°C until analysis (i.e. up to 21 months). Serum levels of adiponectin (µg/ml) were determined with a commercial enzyme-linked immunosorbent assay (R&D, Wiesbaden, Germany). To determine whether storage conditions may have affected adiponectin levels, adiponectin concentrations in aliquots stored for 21 and 33 months at −70°C from 20 patients were measured. Adiponectin levels did not differ significantly between the two timepoints (21 months: 5.6±1.2 (mean±standard deviation) µg/ml versus 33 months: 5.3±1.1 µg/ml; p = 0.45) indicating adequate specimen stability under our storage conditions.

Plasma LDL-C, HDL-C and triglycerides were measured by routine enzymatic methods. Determination of hsCRP levels was performed at the Department of Clinical Chemistry (Campus Grosshadern, University of Munich, Germany).

### Statistical Analysis

Statistical analyses were performed using SAS 9.1 (Cary, NC) software. Data are reported as n or median (interquartile range). Spearman correlation and Wilcoxon two-sample test were used in the bivariate analysis of adiponectin with other variables. A generalized linear regression model was used to assess the association of adiponectin serum levels with atherosclerotic plaque burden, number of calcified plaques, mixed plaques or non-calcified plaques adjusted for age, sex, BMI, diabetes, hypertension, family history of CAD, smoking, LDL-C, HDL-C, triglycerides, hsCRP levels, medical treatment (statin, asa, plavix, marcumar, betablockers, ACE inhibitors, angiotensin-receptor blockers, diuretics, oral anti-diabetic drugs, insulin) and PAT volume - possible confounders of adiponectin levels. All tests were two-tailed with a 0.05 type I error rate.

## Supporting Information

Table S1–S4 Full adjusted model (model 5) for total number of coronary plaques, mixed, non-calcified and calcified plaques
(0.05 MB DOC)
