# Supplementary material for: Low Adiponectin Levels Are an Independent Predictor of Mixed and Non-Calcified Coronary Atherosclerotic Plaques
Source: PLoS One. 2009 Mar 6;4(3):e4733. doi: 10.1371/journal.pone.0004733 (PMC2649379; doi:10.1371/journal.pone.0004733)
Supplement: Table S1–S4 — Full adjusted model (model 5) for total number of coronary plaques, mixed, non-calcified and calcified plaques (0.05 MB DOC) [file pone.0004733.s001.doc]

**Table 1/supplement.** Full adjusted model (model 5) for total number of coronary plaques

Standard Wald 95% Confidence Chi-

Parameter DF Estimate Error Limits Square Pr > ChiSq

Intercept 1 -0.7354 0.4892 -1.6943 0.2234 2.26 0.1328

Adiponectin 1 -0.0360 0.0081 -0.0521 -0.0200 18.92 <.0001

Age 1 0.0326 0.0037 0.0253 0.0399 77.29 <.0001

Sex 1 0.6099 0.0959 0.4220 0.7978 40.47 <.0001

BMI 1 -0.0073 0.0102 -0.0272 0.0126 0.52 0.4705

LDL-cholesterol 1 0.0008 0.0009 -0.0010 0.0026 0.80 0.3712

HDL-cholesterol 1 -0.0068 0.0033 -0.0133 -0.0002 4.14 0.0420

Triglycerides 1 -0.0001 0.0005 -0.0011 0.0009 0.02 0.8801

hsCRP 1 0.0015 0.0212 -0.0401 0.0431 0.01 0.9429

Hypertension 1 0.1403 0.0841 -0.0245 0.3051 2.78 0.0953

Smoking 1 0.0739 0.0826 -0.0881 0.2358 0.80 0.3713

Diabetes 1 -0.4451 0.2493 -0.9338 0.0435 3.19 0.0742

Family history 1 -0.0238 0.0757 -0.1721 0.1245 0.10 0.7533

Asa 1 0.3158 0.0809 0.1573 0.4743 15.25 <.0001

Plavix 1 0.2125 0.0824 0.0511 0.3740 6.65 0.0099

Marcumar 1 0.0957 0.0873 -0.0755 0.2669 1.20 0.2732

Betablocker 1 -0.1912 0.0960 -0.3793 -0.0031 3.97 0.0463

ACE-I, ARB 1 -0.0120 0.0950 -0.1981 0.1741 0.02 0.8994

Statin 1 0.4301 0.0948 0.2443 0.6159 20.58 <.0001

Diuretics 1 -0.0517 0.0792 -0.2069 0.1036 0.43 0.5141

OADs 1 -0.1483 0.2331 -0.6052 0.3086 0.40 0.5247

Insulin 1 0.1618 0.3276 -0.4803 0.8040 0.24 0.6214

Pericardial AT 1 0.0002 0.0004 -0.0006 0.0009 0.19 0.6598

**Table 2/supplement.** Full adjusted model (model 5) for mixed plaques

Standard Wald 95% Confidence Chi-

Parameter DF Estimate Error Limits Square Pr > ChiSq

Intercept 1 -3.3995 1.1730 -5.6985 -1.1005 8.40 0.0038

Adiponectin 1 -0.0871 0.0231 -0.1322 -0.0421 14.51 0.0001

Age 1 0.0370 0.0086 0.0201 0.0539 18.38 <.0001

Sex 1 1.2028 0.2341 0.7439 1.6617 26.39 <.0001

BMI 1 -0.0082 0.0253 -0.0578 0.0415 0.10 0.7474

LDL-cholesterol 1 0.0041 0.0020 0.0003 0.0079 4.37 0.0366

HDL-cholesterol 1 0.0028 0.0078 -0.0124 0.0181 0.13 0.7171

Triglycerides 1 -0.0007 0.0011 -0.0030 0.0015 0.41 0.5206

hsCRP 1 -0.0758 0.0689 -0.2109 0.0593 1.21 0.2715

Hypertension 1 -0.1023 0.1858 -0.4664 0.2617 0.30 0.5817

Smoking 1 -0.2324 0.2000 -0.6244 0.1595 1.35 0.2452

Diabetes 1 0.2909 0.4458 -0.5827 1.1646 0.43 0.5140

Family history 1 -0.1391 0.1697 -0.4718 0.1936 0.67 0.4126

Asa 1 0.2458 0.1871 -0.1209 0.6125 1.73 0.1889

Plavix 1 0.1283 0.1854 -0.2350 0.4917 0.48 0.4888

Marcumar 1 0.1517 0.1996 -0.2394 0.5428 0.58 0.4471

Betablocker 1 -0.3381 0.2154 -0.7603 0.0841 2.46 0.1166

ACE-I, ARB 1 0.1286 0.2326 -0.3273 0.5846 0.31 0.5803

Statin 1 0.9301 0.2253 0.4885 1.3718 17.04 <.0001

Diuretics 1 0.1119 0.1805 -0.2419 0.4657 0.38 0.5353

OADs 1 -0.8133 0.5415 -1.8747 0.2481 2.26 0.1332

Insulin 1 0.3084 0.6287 -0.9239 1.5406 0.24 0.6238

Pericardial AT 1 -0.0010 0.0009 -0.0028 0.0008 1.25 0.2639

**Table 3/supplement.** Full adjusted model (model 5) for non-calcified plaques

Standard Wald 95% Confidence Chi-

Parameter DF Estimate Error Limits Square Pr > ChiSq

Intercept 1 -1.7705 1.0238 -3.7770 0.2360 2.99 0.0837

Adiponectin 1 -0.0760 0.0203 -0.1150 -0.0380 14.94 0.0001

Age 1 0.0144 0.0075 -0.0004 0.0292 3.62 0.0570

Sex 1 0.3245 0.1866 -0.0412 0.6902 3.03 0.0820

BMI 1 0.0104 0.0224 -0.0335 0.0543 0.22 0.6416

LDL-cholesterol 1 0.0048 0.0019 0.0012 0.0085 6.70 0.0096

HDL-cholesterol 1 -0.0049 0.0073 -0.0191 0.0094 0.45 0.5007

Triglycerides 1 0.0014 0.0010 -0.0006 0.0033 1.88 0.1709

hsCRP 1 -0.1740 0.0869 -0.3443 -0.0038 4.01 0.0451

Hypertension 1 0.3038 0.1744 -0.0380 0.6456 3.04 0.0815

Smoking 1 -0.2023 0.1890 -0.5728 0.1681 1.15 0.2844

Diabetes 1 -0.2062 0.4750 -1.1372 0.7249 0.19 0.6643

Family history 1 0.0440 0.1501 -0.2502 0.3382 0.09 0.7696

Asa 1 0.1226 0.1663 -0.2033 0.4484 0.54 0.4610

Plavix 1 0.4738 0.1819 0.1173 0.8304 6.78 0.0092

Marcumar 1 0.4069 0.1691 0.0754 0.7384 5.79 0.0161

Betablocker 1 -0.2508 0.1957 -0.6344 0.1328 1.64 0.2000

ACE-I, ARB 1 0.3407 0.1914 -0.0343 0.7158 3.17 0.0750

Statin 1 0.2153 0.1846 -0.1465 0.5771 1.36 0.2436

Diuretics 1 -0.2265 0.1660 -0.5518 0.0987 1.86 0.1723

OADs 1 0.3065 0.4610 -0.5971 1.2101 0.44 0.5062

Insulin 1 0.8269 0.5619 -0.2745 1.9282 2.17 0.1411

Pericardial AT 1 -0.0006 0.0009 -0.0023 0.0012 0.39 0.5343

**Table 4/supplement.** Full adjusted model (model 5) for calcified plaques

Standard Wald 95% Confidence Chi-

Parameter DF Estimate Error Limits Square Pr > ChiSq

Intercept 1 -3.2311 0.7225 -4.6471 -1.8151 20.00 <.0001

Adiponectin 1 -0.0210 0.0111 -0.0431 0.0010 3.50 0.0612

Age 1 0.0491 0.0057 0.0380 0.0603 74.31 <.0001

Sex 1 0.5536 0.1367 0.2857 0.8215 16.40 <.0001

BMI 1 0.0188 0.0138 -0.0083 0.0460 1.85 0.1736

LDL-cholesterol 1 0.0006 0.0013 -0.0020 0.0033 0.23 0.6293

HDL-cholesterol 1 -0.0100 0.0046 -0.0190 -0.0010 4.73 0.0296

Triglycerides 1 0.0001 0.0007 -0.0013 0.0015 0.01 0.9073

hsCRP 1 0.0666 0.0219 0.0237 0.1095 9.26 0.0023

Hypertension 1 0.1466 0.1203 -0.0893 0.3824 1.48 0.2232

Smoking 1 0.2684 0.1155 0.0421 0.4947 5.40 0.0201

Diabetes 1 -0.9526 0.4354 -1.8060 -0.0992 4.79 0.0287

Family history 1 -0.0500 0.1118 -0.2691 0.1690 0.20 0.6543

Asa 1 0.5020 0.1197 0.2673 0.7367 17.58 <.0001

Plavix 1 0.3092 0.1189 0.0762 0.5423 6.76 0.0093

Marcumar 1 0.1295 0.1297 -0.1247 0.3837 1.00 0.3181

Betablocker 1 -0.0945 0.1417 -0.3721 0.1832 0.44 0.5049

ACE-I, ARB 1 -0.4613 0.1425 -0.7406 -0.1821 10.48 0.0012

Statin 1 0.4190 0.1375 0.1496 0.6884 9.29 0.0023

Diuretics 1 0.1011 0.1173 -0.1289 0.3311 0.74 0.3891

OAD 1 -0.0917 0.3409 -0.7599 0.5765 0.07 0.7879

Insulin 1 -1.0417 0.7465 -2.5049 0.4214 1.95 0.1629

Pericardial AT 1 0.0003 0.0005 -0.0007 0.0014 0.36 0.5488
